# Synthesis of Double-Shelled Hollow Inorganic Nanospheres through Block Copolymer-Metal Coordination and Atomic Layer Deposition

**DOI:** 10.3390/polym11071208

**Published:** 2019-07-19

**Authors:** Nina Yan, Qingbao Guan, Zhiming Yang, Min Feng, Xizhi Jiang, Jun Liu, Lei Xu

**Affiliations:** 1 Institute of Agricultural Facilities and Equipment, Jiangsu Academy of Agricultural Sciences, Nanjing 210014, China; 2 Key Laboratory for Protected Agricultural Engineering in the Middle and Lower Reaches of Yangtze River, Ministry of Agriculture and Rural Affairs, Nanjing 210014, China; 3 State Key Laboratory for Modification of Chemical Fibers and Polymer Materials, International Joint Laboratory for Advanced Fiber and Low-dimension Materials, College of Materials Science and Engineering, Donghua University, Shanghai 201620, China; 4 Jiangsu Bi-gold New Material Stock Co., Ltd., Zhenjiang 212400, China

**Keywords:** block copolymers, self-assembly, metal–ligand coordination, atomic layer deposition, double-shelled hollow structures

## Abstract

Double-shelled hollow (DSH) structures with varied inorganic compositions are confirmed to have improved performances in diverse applications, especially in lithium ion battery. However, it is still of great challenge to obtain these complex nanostructures with traditional hard templates and solution-based route. Here we report an innovative pathway for the preparation of the DSH nanospheres based on block copolymer self-assembly, metal–ligand coordination and atomic layer deposition. Polymeric composite micelles derived from amphiphilic block copolymers and ferric ions were prepared with heating-enabled micellization and metal–ligand coordination. The DSH nanospheres with Fe_2_O_3_ stands inner and TiO_2_ outer the structures can be obtained with atomic layer deposition of a thin layer of TiO_2_ followed with calcination in air. The coordination was carried out at room temperature and the deposition was performed at the low temperature of 80 °C, thus providing a feasible fabrication strategy for DSH structures without destruction of the templates. The cavity and the outer layer of the structures can also be simply tuned with the utilized block copolymers and the deposition cycles. These DSH inorganic nanospheres are expected to find vital applications in battery, catalysis, sensing and drug delivery, etc.

## 1. Introduction

Metal oxides, such as Fe_2_O_3_ and TiO_2_ with low density and high theoretical capacities are widely applied in lithium ion battery [1,2] and photocatalysis [3]. However, their performances are usually limited with low surface area and poor conductivity [3]. To enhance the performance of these materials, hollow structures are proven to be an efficient way as the cavity of the structures can provide extra surface for exposure and decreased electron/ion transport pathways [4]. Meanwhile, compared with hollow structures containing single shell of one composition, double-/multiple-shelled hollow (DSH/MSH) structures with varied compositions are confirmed to broaden their properties or improve their performance [4,5,6]. For example, Zhou and co-workers found that the hollow SnO_2_@TiO_2_ spheres exhibit better electrochemical performance than hollow SnO_2_ spheres [4]. However, fabrication of these complex nanoarchitectures is still challenging and less reported.

The fabrication of hollow structures is usually composed of the utilization of the hard templates, such as SiO_2_ and PS nanospheres, followed with a solution-based route [3,4]. Nowadays, block copolymers are emerging as promising templates for the preparation of numerous hollow structures with functional performance [7], such as metallic compound arrays [8], silicas [9] and carbon materials [10] as they can be self-assembled into diverse well-defined structures due to the two or more thermodynamically incompatible homopolymer chains [11]. Most importantly, block copolymers are considered to have tunable chemical properties, for example, chromophores with hydrophobic interactions [12], quaternization [13] or metal ion coordination with the polyelectrolyte chains [14], thus providing additional functions in the systems.

Compared with solution-based route for the growth of metal oxides, atomic layer deposition (ALD) was applied recently for the production of nanoscopic metal oxides with templates, including TiO_2_, Al_2_O_3_, ZnO, ZrO_2_, etc. [8,15,16,17]. This is a gas-phase deposition technique based on self-limiting reactions between alternately supplied gaseous precursors. Namely, ALD is a simple and reliable pathway for the deposition of monolayer target coatings regardless of the substrates, allowing conformal growth of sub-nanometer precision in coating thickness. Therefore, it is of great simplicity to build double-shelled structures on templates with varied compositions based on ALD strategy. For example, Feng fabricated a hybrid metal oxides with ZnO and TiO_2_ on hydrophilic carbon nanotubes [18]; Wei deposited WO_3_ and Ga_2_O_3_ on SiO_2_/Si substrates in sequence for the fabrication of C_2_H_5_OH sensors with heterostructures [19]. However, even though the deposition of some metal oxides, such as TiO_2_ and Al_2_O_3_ can be carried out at a low temperature of ~80 °C [15,20,21], the deposition of the other target materials usually need to be performed at quite high temperatures. For example, the deposition of Fe_2_O_3_ are usually carried out at the temperature of 250–500 °C [22,23] and ZrO_2_ are deposited at over 160 °C [17,24]. For block copolymer templates, they may be destroyed under these high temperatures, thus ruining the well-defined structures.

Here, we proposed an innovative methodology for the preparation of DSH nanospheres based on block copolymer self-assembly, metal–ligand coordination and atomic layer deposition. The metal ions of ferric were first coordinated with the hydrophilic corona, which will transfer to metal oxides eventually. Afterwards, low-temperature ALD was carried out for the deposition of the second layer of TiO_2_. Both the two steps are aimed at avoiding the decomposition of the block copolymers and the destruction of the nanospheres. Calcination was finally applied for acquiring the DSH structures. The preparation and the morphologies of the micelles and the nanospheres were carefully investigated. The crystal form of the ferric element after calcination was also explored.

## 2. Materials and Methods

### 2.1. Materials

The amphiphilic diblock copolymers of polystyrene-*block*-poly (4-vinyl pyridine) (PS-*b*-P4VP) with three different molecular weights were purchased from Polymer Source Inc., Dorval, Quebec, Canada. The details of these polymers were given in Table 1. Ferric chloride (FeCl_3_) as well as the solvents including acetic acid and ethanol were provided from local suppliers with analytical purity and used as received. Titanium tetrachloride (TiCl_4_) with a purity of 99.99% was obtained from Nanjing University. Deionized water with the conductivity of 9.6 μS cm^−1^ was used in all experiments.

### 2.2. Preparation of Polymeric Composite Micelles by Coordination

The amphiphilic block copolymer micelles were first prepared following the one-step strategy of the heating-enabled micellization in polar solvents [25]. To be specific, designated block copolymers were first mixed with acetic acid with a concentration of 0.2 wt %. The mixture was then transferred into an oven preheated to 110 °C and kept unstirred at this temperature for 17 h. The mixture was subsequently cooled down to room temperature naturally and the milky micellar solution was thus obtained. For the coordination of the micelles with ferric ions, FeCl_3_ was first dissolved in ethanol with a weight percentage of 2 wt %. After ultrasonicated for 10 min, the ferric solution was mixed together with the as-prepared micellar solution with the volume ratio of 1/4. The coordination was carried out for over 24 h and the mixture became yellowish eventually. The as-obtained polymeric composite micelles were then collected from the mixed solutions by centrifugation at 3000 rpm for 2 min and further washed with ethanol four times to remove unreacted FeCl_3_. The composite micelles were redispersed in 2 mL deionized water and then diluted 20 times for further utilization.

### 2.3. Preparation of the DSH Inorganic Nanospheres

50 μL polymeric composite micellar solution was deposited on each piece of substrates, such as pre-cleaned silicon wafers or carbon-coated copper grids, and the substrates were then dried at the temperature of 60 °C for 2 h. After that, the samples were placed in the reaction chamber of a commercialized ALD reactor (Savannah S100, Cambridge NanoTech, USA) preheated to 80 °C. The deposition was carried out at this temperature for different numbers of ALD cycles with a steady N_2_ flow rate of 20 sccm. TiCl_4_ and deionized water were introduced alternatively into the chamber as the Ti and O precursors for TiO_2_ deposition. The typical ALD cycle consists of pulse, exposure and purge of the TiCl_4_ and water vapor with the time of 0.015, 8 and 15 s, respectively. The cycle was repeated for 50, 100, 300 or 500 times. After ALD of TiO_2_, the samples were heated to 540 °C at a rate of 9 °C/min and calcinated at this temperature for 3 h in order to prepare the DSH inorganic nanospheres.

### 2.4. Characterizations

Fourier transformation infrared spectra (FT-IR) of the micelles before and after coordination were characterized by a Nicolet 8700 infrared spectrometer (Thermo Fisher Scientific, Waltham, MA, USA) with the attenuated total reflection mode (ATR). Dynamic light scattering (DLS) was also performed with a particle size analyzer (Zetasizer Nano ZS90, Malvern Panalytical, Malvern, UK) for determining the sizes of the micelles dispersed in water. The morphologies of the samples under different conditions were all examined on silicon wafers with a S-4800 field emission scanning electron microscope (FESEM, Hitachi, Tokyo, Japan) at the accelerating voltage of 5 kV. The samples were first vacuum coated with a thin layer of platinum–palladium alloy for the improvement of conductivity. Transmission electron microscopes (TEM, H-600, Hitachi, Tokyo, Japan & JEM-2100F, JEOL, Tokyo, Japan) were also employed to examine the nanospheres on copper grids before and after ALD. The grids were carefully placed in a small container during the ALD process in order to avoid being pulsed into the vacuum pump. The energy dispersive X-ray spectrometer (EDX) was used to detect the existence of the Fe and Ti elements along with the SEM characterizations and TEM characterizations. X-ray diffraction (XRD) pattern of the nanospheres with 100 TiO_2_ cycles after calcination were obtained from a wide-angle diffractometer with CuKα radiation (λ = 0.154 nm) at a generator voltage of 40 kV and a generator current of 40 mA. The scanning speed and the step were 2.4°/min and 0.02°, respectively.

## 3. Results

### 3.1. The Polymeric Composite Micelles Prepared by Metal–ligand Coordination

**Figure 1:**
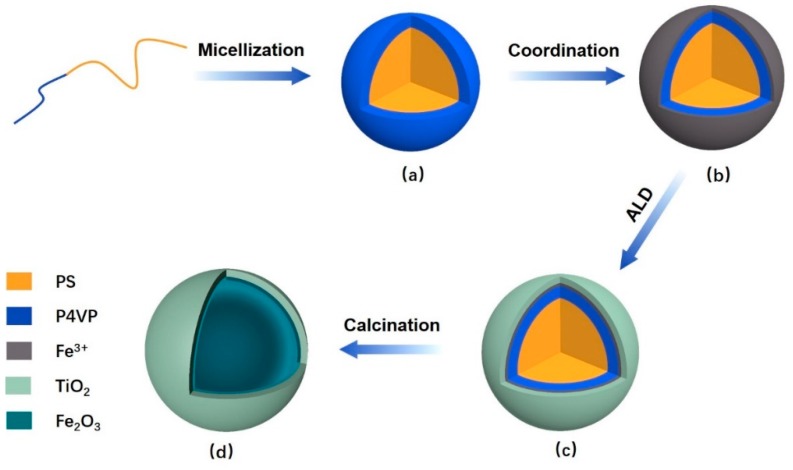
The schematic diagram for the fabrication of double-shelled hollow (DSH) nanospheres through block copolymer-metal coordination and atomic layer deposition (ALD). (**a**) The regular micelles prepared by heating-enabled micellization. (**b**) The polymeric composite micelles after metal–ligand coordination. (**c**) The nanospheres after ALD of TiO_2_. (**d**) DSH inorganic nanospheres after calcination.

**Figure 2:**
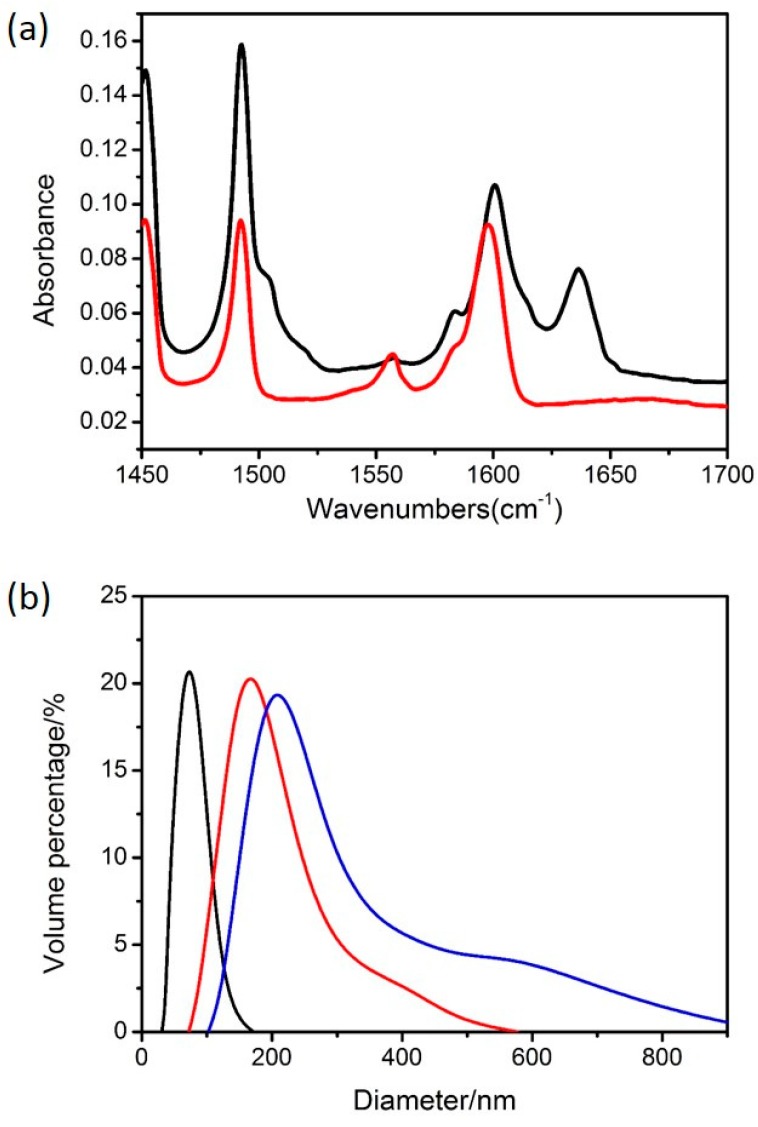
(**a**) The FT-IR spectra of the micelles of PS-*b*-P4VP-2 before (red) and after (black) complexing with ferric ions. (**b**) The size distribution curves of the polymeric composite micelles dispersed in water. The curves correspond to PS-*b*-P4VP-3 (black), PS-*b*-P4VP-2 (red) and PS-*b*-P4VP-1 (blue), respectively.

As the amphiphilic block copolymers are consist of hydrophilic and hydrophobic chains which are covalently linked together, they can self-assembled into either regular or reverse micelles or other nanostructures in selective solvents which are mainly depending on the molecular structures of polymers and the polarity of solvents [26]. For example, the regular micelles with hydrophobic cores and hydrophilic corona can be exclusively formed by dissolution of block copolymers in heating polar solvents [25] while the reverse micelles with reverse constructions are able to be formed with the introduction of nonpolar solvents [27]. Meanwhile, it has been reported that P4VP can function as a ligand to coordinate with metal ions due to the existence of the pyridyl groups [14]. Therefore, amphiphilic block copolymers of PS-*b*-P4VP were employed in this work. The schematic illustration of the preparation route for DSH inorganic nanospheres was depicted in Figure 1. Regular micelles were first prepared with the heating-enabled micellization strategy (Figure 1a). Ferric ions were then introduced for the coordination with the pyridyl groups of the hydrophilic P4VP coronae in order to prepare polymeric composite micelles (Figure 1b). We note that the solution is slightly milky after heating-enabled micellization and it became yellowish after mixing with FeCl_3_, which may represent the occurrence of the coordination. The reactions can also be confirmed with the infrared spectrometry, as shown in Figure 2a. The peaks centering around 1452 and 1493 cm^−1^ should correspond to the characteristic of the phenyl rings of the PS blocks [28]. The peaks at 1558 and 1598 cm^−1^ should be ascribed to the vibration of the C=N and C=C in pyridine rings of P4VP blocks [28,29]. A new peak centering around 1636 cm^−1^ was observed with the polymeric composite micelles, which reveals the coordination between the polymer and the ferric ions [29,30]. Meanwhile, a slight shift (~3–4 wavenumbers) at the wavenumber of 1598 cm^−1^ were also observed with the polymeric composite micelles, thus indicating the interactions of N atom of pyridine rings with the ferric ions [29].

We also examined the size distributions of these composite micelles dispersed in water with DLS, as shown in Figure 2b. According to the results, all of the polymeric composite micelles showed one single size distribution peak and relatively narrow size distributions in the wet state, which indicates the high uniformity of the obtained polymeric composite micelles. The DLS peaks were centered around 210, 157 and 64 nm with the micelles derived from PS-*b*-P4VP-1, PS-*b*-P4VP-2 and PS-*b*-P4VP-3, respectively, representing their mean diameters in the solution. This suggested that the micellar size is strongly dependent on the molecular weights of the employed block copolymers and the micelles are supposed to become larger with the increase of the molecular weights.

**Figure 3:**
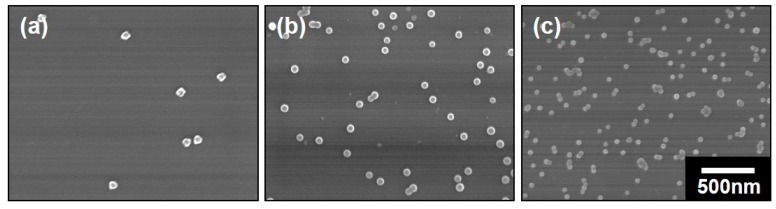
SEM images of the polymeric composite micelles prepared from (**a**) PS-*b*-P4VP-1, (**b**) PS-*b*-P4VP-2 and (**c**) PS-*b*-P4VP-3. All of the images have the same magnification and the scale bar is shown in (**c**).

The morphologies of these polymeric composite micelles were examined with a FESEM and the images were shown in Figure 3. Each kind of composite micelles was uniform with a spherical shape and no aggregation can be observed. This should be attributed to the repulsive forces between the coronae. According to the SEM images, the diameters of the composite micelles prepared from PS-*b*-P4VP-1, PS-*b*-P4VP-2 and PS-*b*-P4VP-3 were determined to be ~75, ~64 and ~46 nm, respectively, which were quite smaller than the values acquired from DLS. This should be owing to the totally different states of the micelles with different characterizations. The samples were supposed to be in wet state with a stretching corona P2VP chains under DLS measurements while the samples were in dry state with shrinking P2VP chains under SEM characterizations, thus leading to the obviously different dimensions. However, we note that the diameters in the dry state also increased with the molecular weights of the used block copolymers, which share similar trend with the values in wet state. Therefore, it is of great simplicity to regulate the micelles with molecular structures of the block copolymers.

### 3.2. Preparation of the Nanospheres by ALD on the Polymeric Composite Micelles

**Figure 4:**
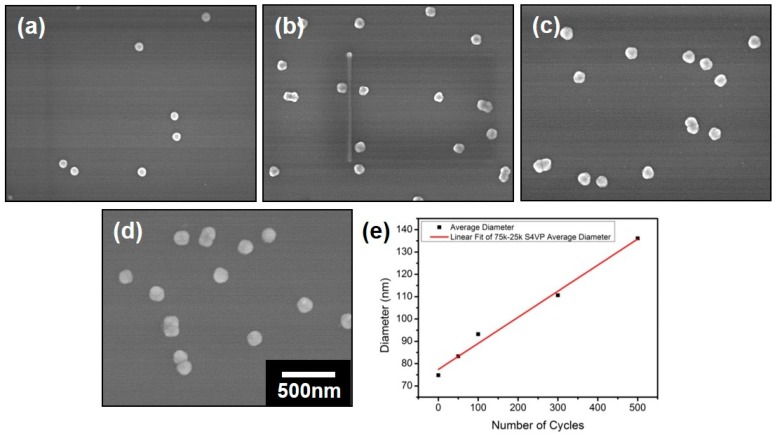
SEM images of the nanospheres of PS-*b*-P4VP-1 subjected to TiO_2_ deposition for (**a**) 50, (**b**) 100, (**c**) 300 and (**d**) 500 cycles, respectively. All of the images have the same magnification and the scale bar is shown in (**d**). (**e**) The plot of the diameters of the nanospheres with the number of ALD cycles.

The as-prepared polymeric composite micelles with coordinated ferric ions were further utilized as the templates for TiO_2_ coatings (Figure 1c). A low temperature deposition was employed in this work in order to avoid the destruction or the degradation of the polymer-based micellar templates. The deposition was carried out at 80 °C for various ALD cycles, which is lower than the glass transition temperatures (*T*_g_) of both the PS and P4VP blocks (*T*_g_^PS^ is 101–106 °C and *T*_g_^P4VP^ is 145–155 °C, respectively, provided by the supplier). This low temperature deposition was also reported elsewhere [20,31,32], thus can be carried out prosperously. Meanwhile, the ferric ions will not be replaced by the titanic precursors, which will be discussed later. The morphologies of the nanospheres derived from PS-*b*-P4VP-1 with various ALD cycles were depicted in Figure 4a–d. We note that the separated micelles are able to maintain their spherical shapes after low temperature deposition regardless of the ALD cycles, thus verified the feasibility of the deposition. The diameters of the nanospheres had a visible increment with the increase of the ALD cycles. To further understand the relationship between the diameters of the nanospheres and the number of ALD cycles, the plot of the mean diameters of the nanospheres with the number of cycles was given in Figure 4e. As discussed in Figure 3a, the diameter of the composite micelles was measured to be ~75 nm before deposition. After TiO_2_ deposition for 50, 100, 300 and 500 cycles, the nanospheres were determined to be ~83, ~93, ~109 and ~136 nm, respectively, as listed in Table 2. According to the fitted curves, the diameters of the nanospheres were increased with the number of ALD cycles at an average rate of ~1.16 Å per cycle. However, the growth of the nanospheres with the cycle numbers was nonlinear in fact. The deposition rate is observed to be faster at the first 100 cycles with a mean value of ~1.80 Å than that for the later 400 cycles with the value of ~1.08 Å, which may probably be owing to the varied diffusion rate of the precursors. In the initial stage, the intersphere distance is large enough for the rapid diffusion of the precursor vapors. However, with the increment of the ALD cycles, the nanospheres were getting larger with the intersphere space getting reduced correspondingly, thus increased the diffusion resistance of the ALD precursors and lower the deposition rate. The varied deposition substrates may also be an important factor for the changed deposition rate. In the initial stage, the precursors of TiCl_4_ was supposed to coordinate first with the unreacted superficial free pyridyl groups of P4VP coronae, thus harvested Ti precursors rapidly. Further exposure to the precursors led to the growth of TiO_2_ on the preformed TiO_2_ thin layer, which may result in the different growth rate of TiO_2_ eventually.

The morphologies and the plot of nanospheres derived from PS-*b*-P4VP-2 and PS-*b*-P4VP-3 with different ALD cycles were also shown in Figures S1 and S2. The diameters of these nanospheres were also listed in Table 2. We found that the diameters of both kinds of nanospheres have an increment with the ALD cycles, which is similar to that of the nanospheres prepared from PS-*b*-P4VP-1. The average growth rate for the deposition on PS-*b*-P4VP-2 and PS-*b*-P4VP-3 are ~1.43 Å and ~1.29 Å per cycle, respectively, which is both close to the growth rate of the deposition on PS-*b*-P4VP-1. However, differ from the nonlinear increase with the PS-*b*-P4VP-1, the growth rate for the deposition on nanospheres with PS-*b*-P4VP-2 and PS-*b*-P4VP-3 are both found to be nearly linear increased with the number of cycles. This may probably be ascribed to the smaller diffusion rate change of the precursors with initial relatively smaller nanospheres and correspondingly broader intersphere distance. Moreover, the thickness of TiO_2_ layer on composite micelles of PS-*b*-P4VP-1 is calculated to be only ~4 nm with 50 ALD cycles and ~9 nm with 100 ALD cycles. The TiO_2_ layer was increased to ~17 and ~33 nm with 300 and 500 cycles of ALD. The thickness of the TiO_2_ layer on PS-*b*-P4VP-2 and PS-*b*-P4VP-3 were both nearly equal to these values under same ALD cycles with a small error of only 2–3 nm. Therefore, it is also a simple way to tune the thickness of the TiO_2_ layer by changing the ALD cycles. Meanwhile, the nanospheres prepared with lower molecular weight were determined to be smaller than those prepared with higher molecular weight under the same deposition cycles.

**Figure 5:**
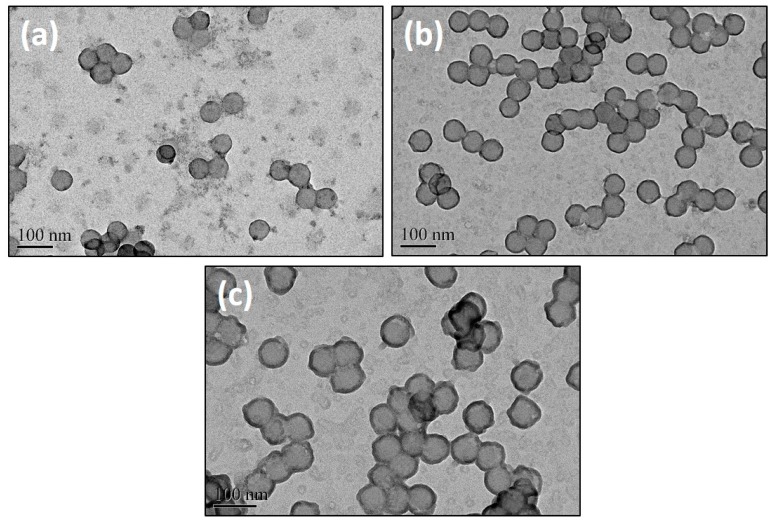
TEM images of the nanospheres of PS-*b*-P4VP-1 subjected to TiO_2_ deposition for (**a**) 0, (**b**) 50 and (**c**) 100 cycles, respectively.

The structures of the nanospheres derived from PS-*b*-P4VP-1 with different ALD cycles were also investigated by TEM carefully. As demonstrated in Figure 5a, an ultrathin dark layer with the thickness of ~3 nm around the polymeric composite micelles was observed without ALD. Considering the similar electron density of PS and P4VP chains, the dark layer should only be ascribed to the presence of the ferric ions as Fe element have higher electron density than that of the polymers [15]. This result illustrated the successful coordination of the ferric ions with the pyridyl groups of the coronae P4VP chains. After ALD of TiO_2_ for 50 cycles (Figure 5b), the thickness of the dark layer around nanospheres increased to ~6 nm and it further increased to ~10 nm after 100 cycles (Figure 5c). Therefore, the TiO_2_ layer is determined to be 3 and 7 nm with 50 and 100 cycles. Considering the different principles between SEM and TEM characterizations, these values were in good agreement with the results obtained from SEM characterizations. The EDX mapping of the PS-*b*-P4VP-1 nanospheres with 50 ALD cycles was also given in Figure S3. The Fe and Ti elements were both observed while Fe element mainly existed closer to the center of the nanospheres than Ti element. This verified the double-shelled structures after TiO_2_ deposition with Fe element exists inner and Ti element outer the shell of the nanospheres. Meanwhile, the precursors of TiCl_4_ will not replace the coordinated ferric ions with the ALD process. The structures of the nanospheres derived from PS-*b*-P4VP-3 with different ALD cycles were also investigated and the results were shown in Figure S4. The dark layer around the polymeric composite micelles was also observed and the size of the nanospheres of PS-*b*-P4VP-3 was found to be much smaller than that of PS-*b*-P4VP-1 with the same ALD cycles.

### 3.3. The Preparation of the DSH Inorganic Nanospheres

**Figure 6:**
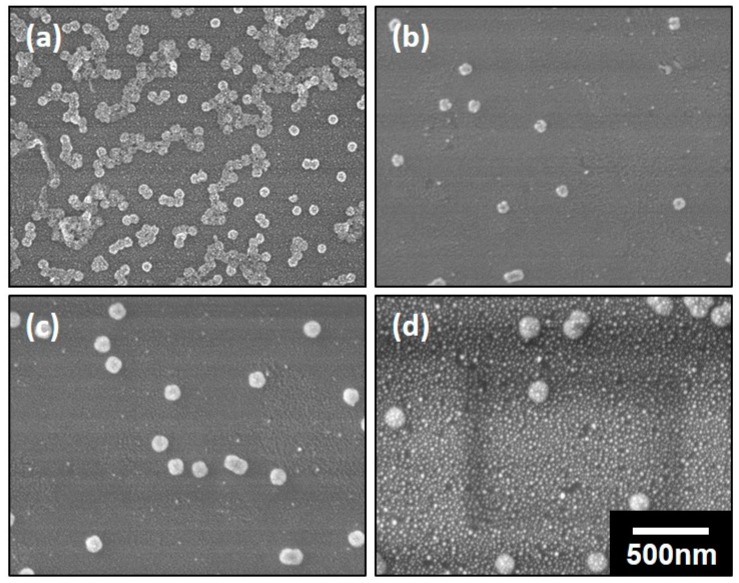
SEM images of the nanospheres of PS-*b*-P4VP-1 subjected to TiO_2_ deposition for (**a**) 50, (**b**) 100, (**c**) 300 and (**d**) 500 cycles followed by calcination. All of the images have the same magnification and the scale bar is shown in (**d**).

In order to prepare DSH structures, the as-deposited nanospheres were calcinated in air at 540 °C for 3 h. After calcination, the block copolymers were degraded while the coordinated ferric ions were supposed to be transformed into a thin layer of iron oxide and the outer TiO_2_ layer kept the shell skeleton, leading to the hollow structures. It is also supposed that the calcination leads to the formation of crystalline TiO_2_ while the TiO_2_ produced at low temperature was usually weakly crystallized or even amorphous [33]. The morphologies of the nanospheres of PS-*b*-P4VP-1 subjected to TiO_2_ deposition for different ALD cycles after calcination was depicted in Figure 6. The nanospheres can maintain their spherical structures without any destruction after calcination even if the TiO_2_ layer was as thin as only ~4 nm with 50 ALD cycles. Meanwhile, with the increment of the ALD cycles, the diameters of the nanospheres after calcination increased accordingly. For example, the diameter of the nanospheres was determined to be ~62 nm with 50 cycles (Figure 6a) and it can be further increased to ~73, ~105 and ~135 nm with 100, 300 and 500 cycles (Figure 6b–d). However, it is surprising that the diameters of the nanospheres with lower ALD cycles are decreased when compared with the diameters before calcination. As the ALD carried out for over 300 cycles, the diameters of the nanospheres were nearly the same either before or after calcination, suggesting no deformation of the structures. Therefore, it is believed that the nanospheres were partly shrinked as the thickness of the TiO_2_ layer is lower than ~17 nm. The morphologies of the nanospheres of PS-*b*-P4VP-2 after calcination were also shown in Figure S5. The diameters of nanospheres were determined to be ~65 nm with 50 ALD cycles, which is smaller than the value obtained from SEM characterizations, indicating a shrinkage of the nanospheres after calcination. However, the diameter was supposed to be ~78 nm with 100 ALD cycles, which is close to the result from SEM characterizations. Therefore, nanospheres derived from lower molecular weights were able to maintain their structures without any deformation with thinner TiO_2_ layers, for example, ~7 nm.

**Figure 7:**
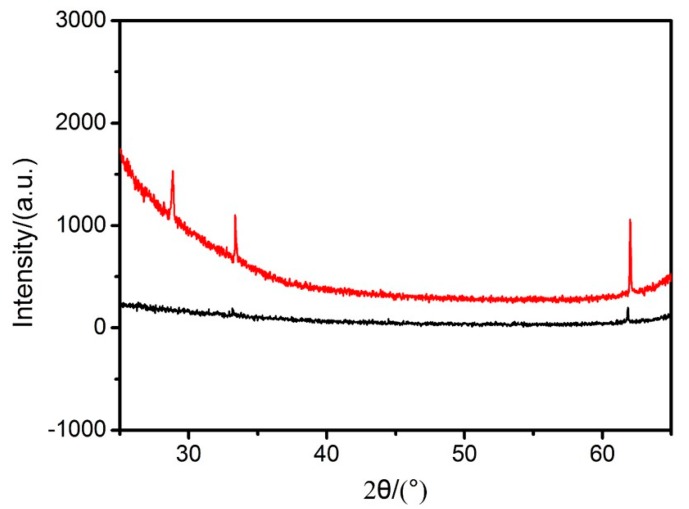
Comparison of the XRD patterns of silicon wafers (black) with the hollow nanospheres of PS-*b*-P4VP-1 after calcination (red).

To further confirm the existence of the iron and titanium elements after calcination, EDX was utilized for the characterizations of the PS-*b*-P4VP-1 samples and the results were summarized in Table 3. After calcination, the block copolymers were fully degraded as the PS-*b*-P4VP can be fully removed at the temperature of over 300 °C [15]. As evidenced by the results, the iron and titanium elements were still existed in the samples even though their amounts were finite. Considering the nanospheres were sporadically coated on the silicon wafers with a quite thin silica layer existed on the surface, it is reasonable to understand that the amount of Fe and Ti elements are much lower than those of Si and O elements. Meanwhile, as the calcination was carried out in air, the ferric ions coordinated on the micelles were considered to be transformed into a thin layer of iron oxide. To further confirm the crystal form of the Fe element, the XRD pattern of the hollow nanospheres of PS-*b*-P4VP-1 with 100 ALD cycles was exhibited in Figure 7. XRD analysis showed that the DSH nanospheres present a peak centering at 33°, which is coincident with a characteristic peak of α-Fe_2_O_3_. Therefore, it indicated a transition of ferric ions to a moderate crystallinity of α-Fe_2_O_3_ after calcination in air and it is credible that the double-shelled polymeric nanospheres are transferred to the DSH inorganic nanospheres with Fe_2_O_3_ exists inner and TiO_2_ outer the hollow spherical structures.

## 4. Conclusions

We have demonstrated an innovative strategy for producing DSH inorganic nanospheres with the block copolymer micelles being employed as the template. In this strategy, amphiphilic block copolymers were first self-assembled into regular micelles with heating-enabled micellization. With the introduction of the metal–ligand coordination, ferric ions were supposed to be coordinated on the coronae of the regular micelles, forming polymeric composite micelles. The composite micelles were then deposited with a thin layer of TiO_2_ by ALD and the DSH inorganic nanospheres can be obtained with Fe_2_O_3_ existing inner and TiO_2_ outer the hollow spherical structures after calcination in air. The coordination was carried out at room temperature and the ALD process was performed at the low temperature of 80 °C. Therefore, this strategy provides a feasible strategy for preparing double-shelled structures without destruction of the block copolymer templates with some designated metal oxide which must be deposited with quite high temperatures. The hollow structures can also be finely tuned with the utilized molecular structures of the block copolymers and the ALD cycles. These DSH inorganic nanospheres are expected to find vital applications in battery, catalysis, sensing and drug delivery, etc.

## Figures and Tables

**Table 1:** The molecular weights and the polydispersity of the block copolymers.

Block Copolymers	*M*_n_^PS^	*M*_n_^P4VP^	Polydispersity
PS-*b*-P4VP-1	75,000	25,000	1.09
PS-*b*-P4VP-2	50,000	17,000	1.15
PS-*b*-P4VP-3	23,000	4,500	1.10

**Table 2:** The diameters of the nanospheres derived from different block copolymers with varied ALD cycles ^1^.

Cycles	PS-*b*-P4VP-1	PS-*b*-P4VP-2	PS-*b*-P4VP-3
0	75 ± 5	64 ± 4	46 ± 4
50	83 ± 4	71 ± 3	55 ± 4
100	93 ± 7	77 ± 4	61 ± 5
300	109 ± 5	106 ± 5	85 ± 6
500	136 ± 6	135 ± 5	112 ± 6

^1^ The unit is nm.

**Table 3:** EDX analysis of the hollow nanospheres of PS-*b*-P4VP-1 after ALD and calcination.

Element	Weight %	Atomic %
O K	6.04	10.16
Si K	93.39	89.54
Ti K	0.32	0.18
Fe K	0.25	0.12
Totals	100.00	

## Supplementary Materials

The following are available online at https://www.mdpi.com/2073-4360/11/7/1208/s1, Figure S1: SEM images of the nanospheres of PS-*b*-P4VP-2 subjected to TiO_2_ deposition for (a) 50, (b) 100, (c) 300 and (d) 500 cycles, respectively. All of the images have the same magnification and the scale bar is shown in (d). (e) The plot of the diameters of the nanospheres with the number of ALD cycles, Figure S2: SEM images of the nanospheres of PS-*b*-P4VP-3 subjected to TiO_2_ deposition for (a) 50, (b) 100, (c) 300 and (d) 500 cycles, respectively. All of the images have the same magnification and the scale bar is shown in (d). (e) The plot of the diameters of the nanospheres with the number of ALD cycles, Figure S3: EDX mapping of the micelles of PS-*b*-P4VP-1 subjected to TiO_2_ deposition for 50 cycles, Figure S4: TEM images of the nanospheres of PS-*b*-P4VP-3 subjected to TiO_2_ deposition for (a) 0, (b) 50 and (c) 100 cycles, respectively, Figure S5: SEM images of the nanospheres of PS-*b*-P4VP-2 subjected to TiO_2_ deposition for (a) 50, (b) 100, (c) 300 and (d) 500 cycles followed with calcination. All of the images have the same magnification and the scale bar is shown in (d).
