# Supplementary material for: Synthesis of Double-Shelled Hollow Inorganic Nanospheres through Block Copolymer-Metal Coordination and Atomic Layer Deposition
Source: Polymers (Basel). 2019 Jul 19;11(7):1208. doi: 10.3390/polym11071208 (PMC6681095; doi:10.3390/polym11071208)
Supplement: Supplementary file 1 [file polymers-11-01208-s001.pdf]

Supporting information

for

Synthesis of double-shelled hollow inorganic nanospheres through block copolymer-metal coordination and atomic layer deposition

Nina Yan[1,2], Qingbao Guan[3], Zhiming Yang[4], Min Feng[1,2], Xizhi Jiang[1,2], Jun Liu[1,2] and Lei Xu[1,2,]*

[1] Institute of Agricultural Facilities and Equipment, Jiangsu Academy of Agricultural Sciences, *Nanjing 210014*, *Jiangsu, China*

[2] Key Laboratory for Protected Agricultural Engineering in the Middle and Lower Reaches of Yangtze River, Ministry of Agriculture and Rural Affairs, Nanjing 210014, Jiangsu, China

[3] State Key Laboratory for Modification of Chemical Fibers and Polymer Materials, International Joint Laboratory for Advanced Fiber and Low-dimension Materials, College of Materials Science and Engineering, Donghua University, *Shanghai 201620, China*

[4] Jiangsu Bi-gold New Material Stock Co., Ltd, *Zhenjiang 212400, Jiangsu, China*

** Corresponding Authors*

E-mail: xulei@jaas.ac.cn (L. Xu)

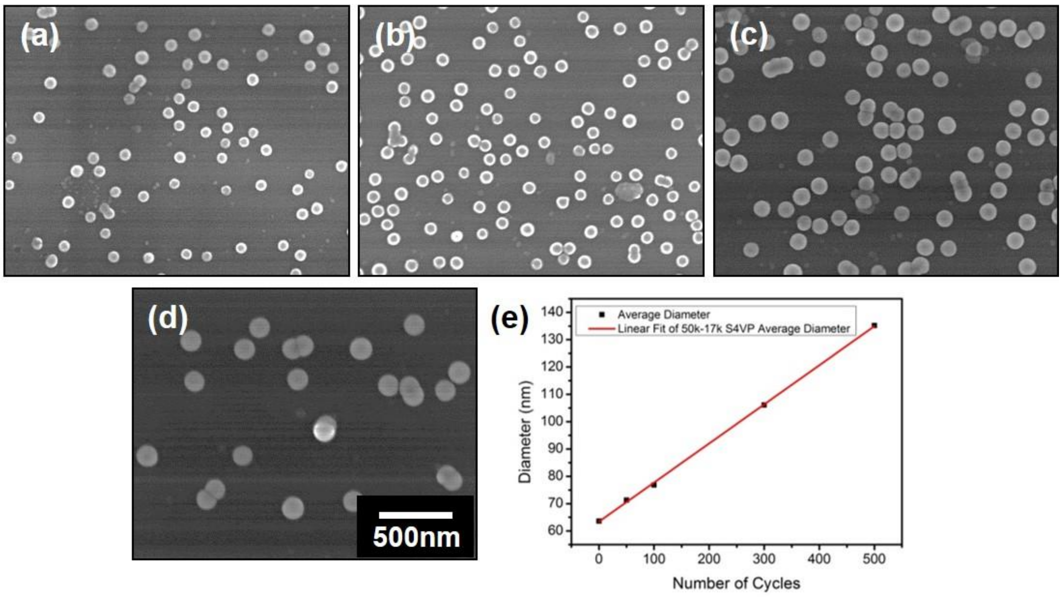

Figure S1. SEM images of the nanospheres of PS-*b*-P4VP-2 subjected to TiO_2 deposition for (a) 50, (b) 100, (c) 300 and (d) 500 cycles, respectively. All of the images have the same magnification and the scale bar is shown in (d). (e) The plot of the diameters of the nanospheres with the number of ALD cycles.

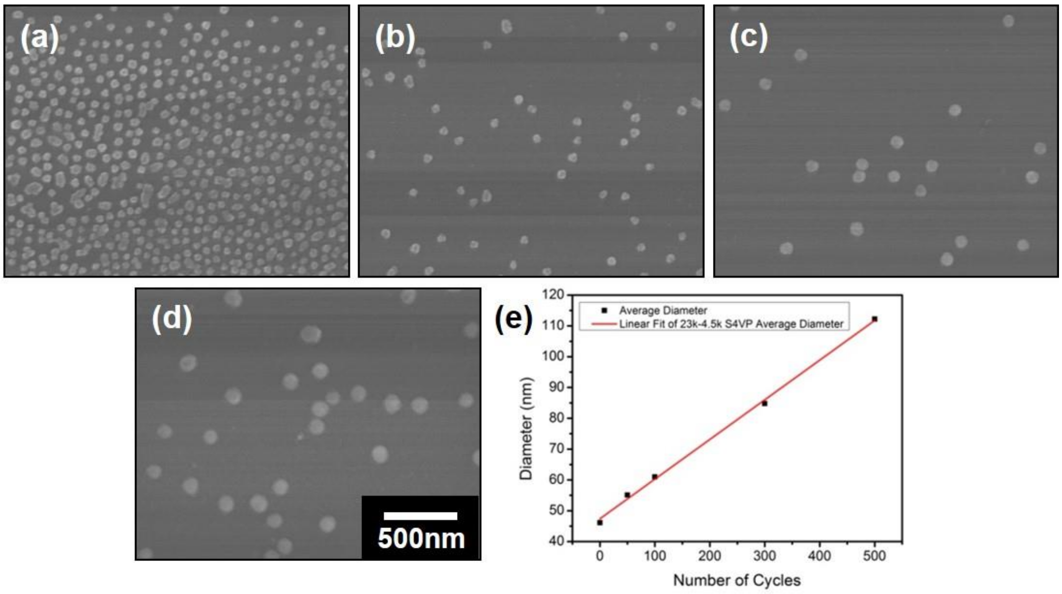

Figure S2. SEM images of the nanospheres of PS-*b*-P4VP-3 subjected to TiO_2 deposition for (a) 50, (b) 100, (c) 300 and (d) 500 cycles, respectively. All of the

images have the same magnification and the scale bar is shown in (d). (e) The plot of the diameters of the nanospheres with the number of ALD cycles.

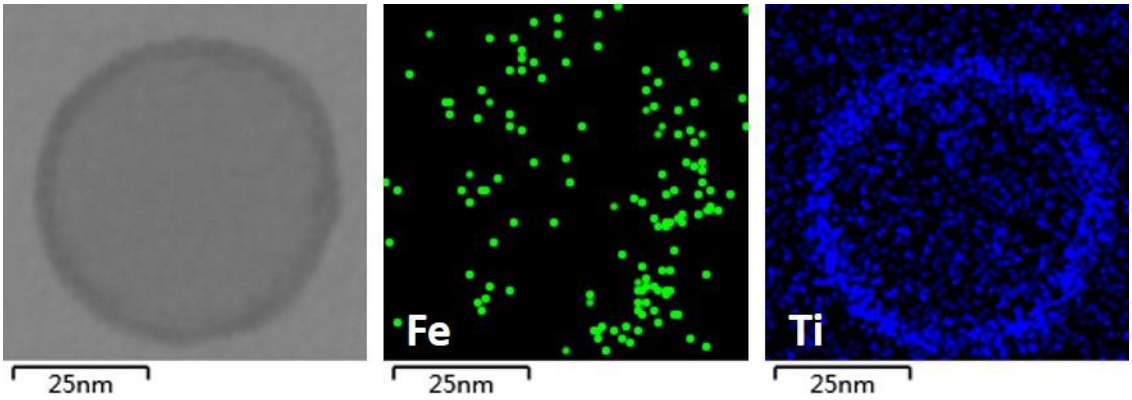

Figure S3. EDX mapping of the micelles of PS-*b*-P4VP-1 subjected to TiO_2 deposition for 50 cycles.

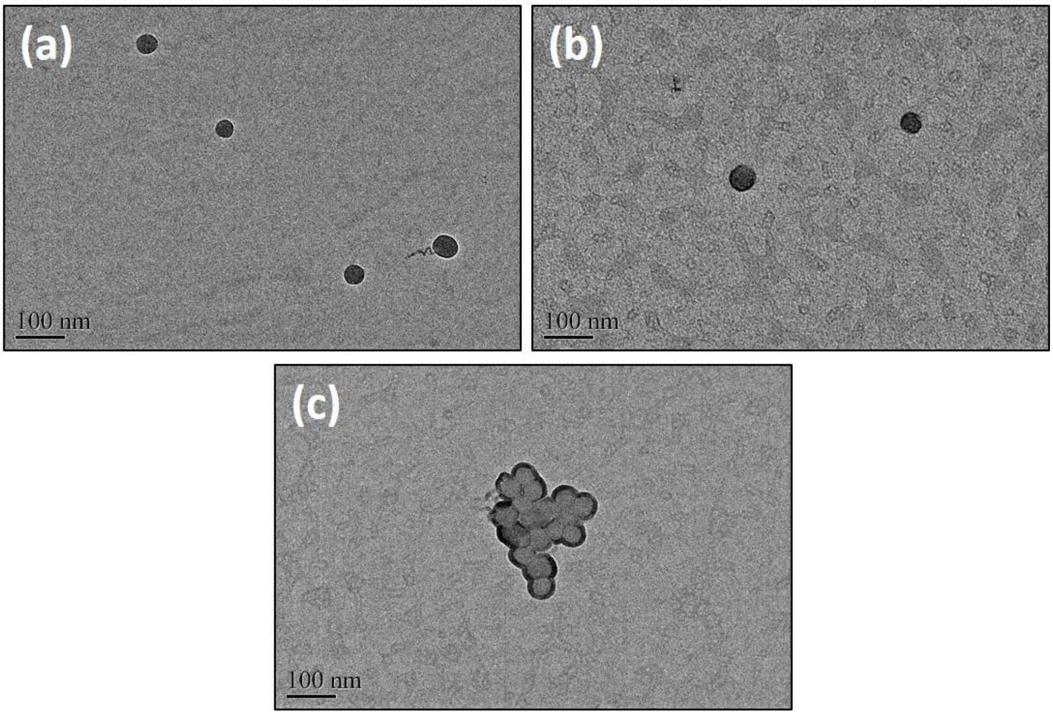

Figure S4. TEM images of the nanospheres of PS-*b*-P4VP-3 subjected to TiO_2 deposition for (a) 0, (b) 50 and (c) 100 cycles, respectively.

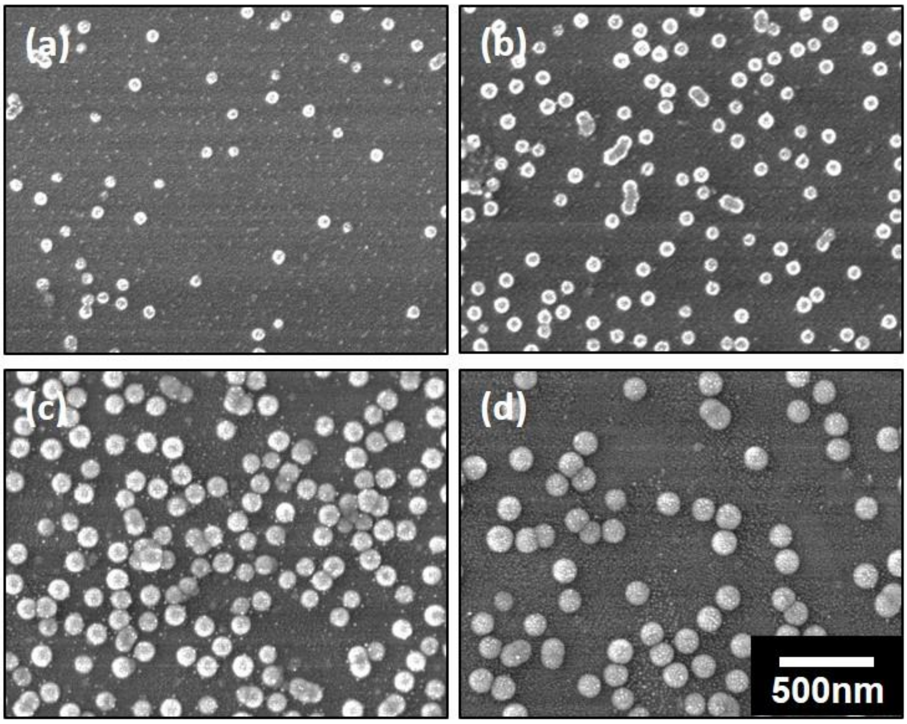

Figure S5. SEM images of the nanospheres of PS-*b*-P4VP-2 subjected to TiO_2 deposition for (a) 50, (b) 100, (c) 300 and (d) 500 cycles followed with calcination. All of the images have the same magnification and the scale bar is shown in (d).
